# Comparison of global myocardial strain assessed by cardiovascular magnetic resonance tagging and feature tracking to infarct size at predicting remodelling following STEMI

**DOI:** 10.1186/s12872-016-0461-6

**Published:** 2017-01-05

**Authors:** Abhishek M. Shetye, Sheraz A. Nazir, Naveed A. Razvi, Nathan Price, Jamal N. Khan, Florence Y. Lai, Iain B. Squire, Gerald P. McCann, Jayanth R. Arnold

**Affiliations:** 1 Department of Cardiovascular Sciences, University of Leicester, Glenfield Hospital, Groby Road, Leicester, LE3 9QF UK; 2 Oxford University Hospitals NHS Trust, Oxford, OX3 9DU UK; 3 Ipswich Hospital NHS trust, Ipswich, IP4 5PD UK; 4 Leeds Institute of Cardiovascular and Metabolic Medicine (LICAMM), University of Leeds, Leeds, LS2 9JT UK

**Keywords:** Cardiac magnetic resonance, Tagging, Feature tracking, Strain, Remodelling, ST-elevation myocardial infarction

## Abstract

**Background:**

To determine if global strain parameters measured by cardiovascular magnetic resonance (CMR) acutely following ST-segment Elevation Myocardial Infarction (STEMI) predict adverse left ventricular (LV) remodelling independent of infarct size (IS).

**Methods:**

Sixty-five patients with acute STEMI (mean age 60 ± 11 years) underwent CMR at 1–3 days post-reperfusion (baseline) and at 4 months. Global peak systolic circumferential strain (GCS), measured by tagging and Feature Tracking (FT), and global peak systolic longitudinal strain (GLS), measured by FT, were calculated at baseline, along with IS. On follow up scans, volumetric analysis was performed to determine the development of adverse remodelling – a composite score based on development of either end-diastolic volume index [EDVI] ≥20% or end-systolic volume index [ESVI] ≥15% at follow-up compared to baseline.

**Results:**

The magnitude of GCS was higher when measured using FT (−21.1 ± 6.3%) than with tagging (−12.1 ± 4.3; *p* < 0.001 for difference). There was good correlation of strain with baseline LVEF (r 0.64–to 0.71) and IS (*ρ* -0.62 to–0.72). Baseline strain parameters were unable to predict development of adverse LV remodelling. Only baseline IS predicted adverse remodelling – Odds Ratio 1.05 (95% CI 1.01–1.10, *p* = 0.03), area under the ROC curve 0.70 (95% CI 0.52–0.87, *p* = 0.04).

**Conclusion:**

Baseline global strain by CMR does not predict the development of adverse LV remodelling following STEMI.

## Background

Adverse left ventricular (LV) remodelling is associated with poor outcome following ST-segment Elevation Myocardial Infarction (STEMI) [1–3]. Therefore, early recognition of at risk patients may enable targeted therapeutic intervention to attenuate adverse remodelling, and thereby reduce the progression to heart failure, and improve clinical outcome.

Myocardial strain describes the relative change in length of myocardial segments and provides an objective measure of LV function [4]. We have previously conducted a systematic review of seven studies that showed global longitudinal strain (GLS) as measured by speckle-tracking echocardiography predicts clinical outcome and LV remodelling following STEMI [5]. Infarct size (IS) has been shown to be a powerful predictor of adverse remodelling and prognosis following STEMI [6, 7]. However, IS cannot be quantified using routine echocardiography. Another disadvantage with echocardiography is that strain analysis may be hampered by variable image quality and the limited number of short axis views that are acquired [8], precluding the reliable assessment of global circumferential strain (GCS).

CMR is the gold standard non-invasive technique for the assessment of LV volumes and IS quantification [9, 10]. Myocardial tissue tagging has traditionally been regarded as the reference method for the quantification of peak systolic strain [11, 12]. Feature Tracking (FT) is a novel post-processing strain quantification technique that can be performed on routinely acquired steady-state free precession cine sequences. This avoids the difficulties of tagging, which requires additional image acquisitions with prolonged breath holding and time-consuming post-processing analysis [13, 14]. In a previous study of 24 patients following STEMI, FT was shown to be more robust and quicker to analyse, providing stronger correlation with IS and superior intra- and inter-observer variability when compared with tagging [14].

In one study of 74 patients following STEMI [15], GCS by FT predicted global functional recovery (left ventricular ejection fraction [LVEF] >50%) at follow-up but its utility in identifying patients who subsequently develop adverse remodelling was not demonstrated. Furthermore, there was no adjustment for IS, and strain as measured by FT was not compared with the established standard of tagging.

To date, there have been no reports assessing whether CMR-measured global strain is associated with adverse LV remodelling independent of IS. The primary aim of this study was to determine whether global strain parameters as assessed by CMR-tagging and FT could predict the development of adverse LV remodelling and whether they provide any incremental value to IS.

## Methods

### Study population

In a previously published study assessing the prevalence and extent of microvascular obstruction following STEMI, we recruited patients presenting to a single, regional cardiac centre in the UK with a first STEMI between January 2010 and December 2012 – the inclusion and exclusion criteria have been previously defined [16]. For the present study, the functional data derived from this earlier study were analysed. Baseline CMR was performed 1–3 days post reperfusion, with follow-up CMR, 4 months after admission. The study was conducted according to the Declaration of Helsinki, was approved by the local research ethics committee (Derbyshire Research Ethics Committee, **09/H0401/21**) and all patients provided written informed consent.

### Imaging protocol

**Fig. 1:**
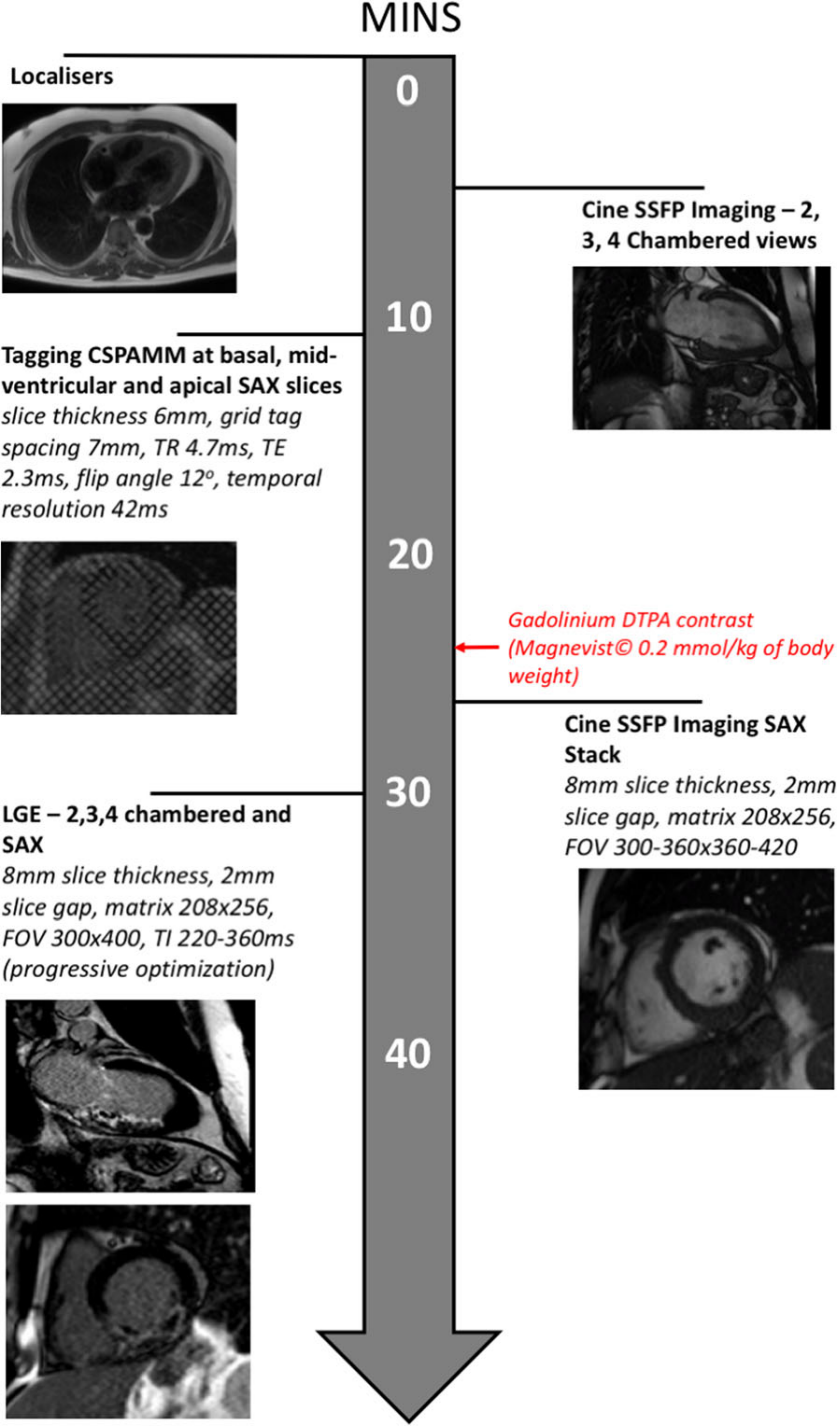
Protocol for CMR scan. Abbreviations:CSPAMM (Complementary Spatial Modulation of Magnetisation), FOV Field of View, LGE (Late Gadolinium Enhancement), SAX (Short Axis), SSFP (Steady State Free Precession), TE Echo Time, TI Inversion Time, TR Repetition Time

CMR was performed on a 1.5 T scanner (Siemens Avanto, Erlangen, Germany) using a 6-channel phased-array cardiac receiver coil – see Fig. 1 for study imaging protocol for CMR scan (common at both baseline and follow-up). Cine and late gadolinium enhancement imaging were performed as previously described [16]. Three equidistant tagging short axis (SAX) cine images were acquired using complementary spatial modulation of magnetisation (CSPAMM) at base, mid-ventricle and apex.

### Image analysis

All images were anonymised and, following completion of the study, were analysed offline by experienced operators blinded to all clinical information. Volumetric analysis and IS quantification were performed at the time of the original study whilst global strain assessment by tagging and FT was performed post-hoc after the original study had been completed.

### Myocardial tagging

**Fig. 2:**
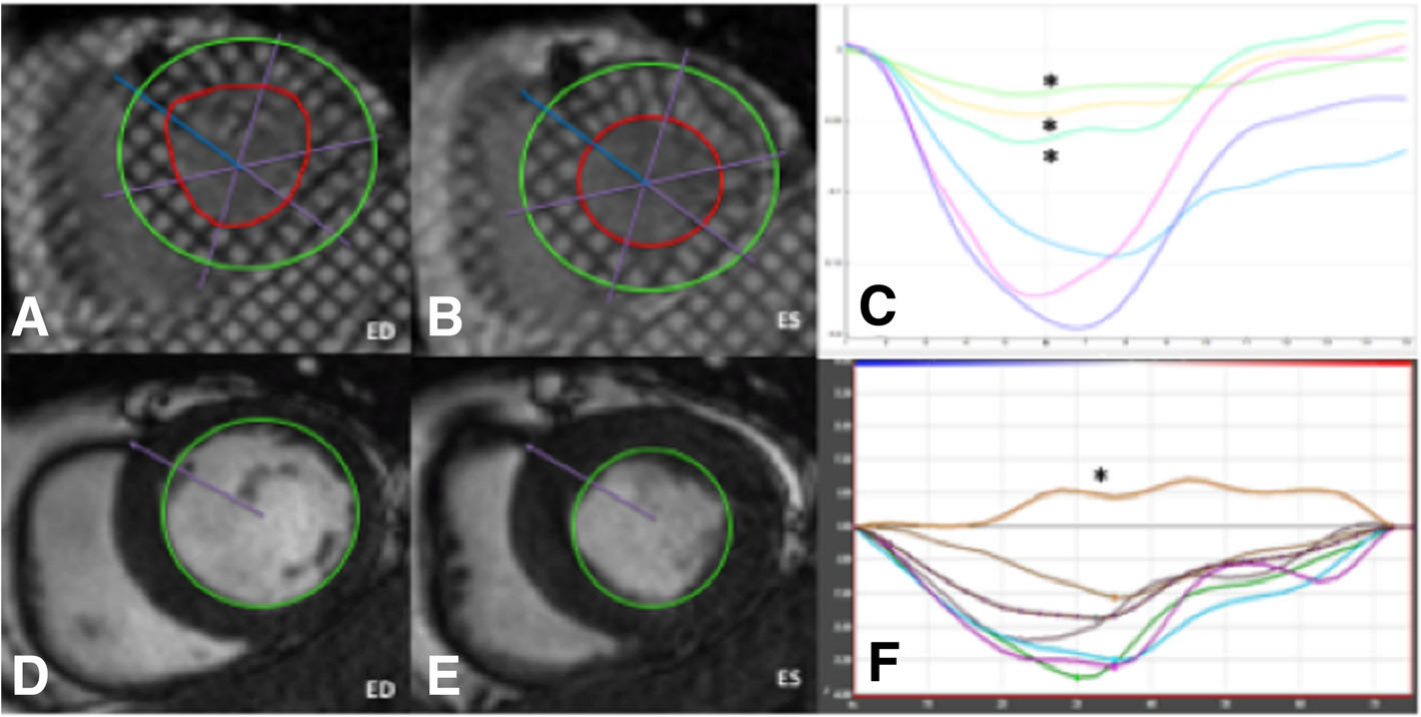
Comparison of strain analysis by tagging and feature tracking (FT) at short axis (SAX). (**a**, **b**) Tagged complementary spatial modulation of magnetisation (CSPAMM) basal short axis slice shown with endocardial and epicardial contours at *end-diastole* (*ED*) *end-systole* (*ES*) in a patient following inferior MI. (**c**) The resultant peak systolic circumferential strain curve at each segment with severely hypokinetic segments denoted by an (*). (**d**, **e**) Feature Tracking (FT) on cine Steady State Free Precession (SSFP) SAX slice with endocardial borders defined is shown at *ED* (**d**) and *ES* (E). (**f**) Segmental peak systolic circumferential strain curve by FT with dyskinetic segment denoted by an asterisk (*)

Strain was evaluated on SAX tagging images (NP) acquired at baseline using the SinMOD algorithm – *InTag* post-processing plugin (Creatis, Lyon, France) for *OsiriX* (Pixmeo, Switzerland) [13] – see Fig. 2. GCS was derived as an average of the values assessed at the three SAX slices as previously described [17]. Tagging images were not acquired in long axis views and consequently, only circumferential strain analysis was performed.

### Feature tracking (ft)

Strain analysis on FT was performed using the *Diogenes Feature Tracking V6.3* (TomTec Imaging Systems, Munich, Germany) (AS) as previously described [14, 17, 18]. Briefly, endocardial contours were defined at end-diastole on SAX and long axis cine steady state free precession images and propagated – see Fig. 2. To determine GCS, three equidistant SAX slices that best represented the base, mid-ventricle and apex were selected from the SAX stack similar to those used for tagging. To determine GLS, analysis was performed using the 2, 3 and 4-chamber long axis views.

### Volumetric analysis & infarct size quantification

Volumetric, functional and IS assessment were performed using *QMass V7.1* (Medis, Leiden, Netherlands) (NAR and JNK). All volumes were indexed to Body Surface Area. IS was quantified semi-automatically using the full-width half-maximum technique as previously described [19]. Adverse LV remodelling was calculated as a composite score based on development of either a relative increase in end-systolic volume index of 15% or a relative increase in end-diastolic volume index of 20% at follow-up compared with baseline [1, 20]

### Statistical analysis

Normality was assessed using the Shapiro-Wilk test, histograms and Q-Q plots. Normally distributed data are expressed as mean ± SD whilst non-normally distributed data are shown as median (interquartile range). Comparison of normally distributed data was performed using paired *t*-test, and non-normally distributed data, using Mann–Whitney *U*-test, and categorical data, using Chi-Squared test. Correlation between parameters was assessed using either Pearson’s correlation coefficient (r) or Spearman’s rank coefficient (ρ) where appropriate.

Logistic regression was performed to assess predictors of the LV remodelling parameters. Receiver operator characteristic curve analysis was performed for all significant predictors of adverse LV remodelling. All values with *p* < 0.05 were considered statistically significant. Statistical analysis was performed using *SPSS version 22.0* (Chicago, IL)

## Results

### Baseline characteristics

Table 1 summarised the baseline characteristics of the study population. Sixty-five patients underwent baseline and follow-up CMR scans. Patients were treated as follows: primary PCI (*n* = 39, 60%), thrombolysis (*n* = 13, 20%), rescue PCI (*n* = 8, 12%) and late-PCI (*n* = 5, 8%). Thrombolysis was performed with tissue plasminogen activator analogues where facilities for primary PCI were not available within a two-hour period of symptom-onset. Rescue PCI was performed in patients in whom ST-segment resolution of >50% post-thrombolysis was not achieved. Late PCI describes primary PCI >12 h after symptom onset in the presence of ECG and/or clinical evidence of continuing ischaemia.

**Table 1:** Key patient characteristics

Demographics			
Age, years	59.5 ± 11.0		
Male sex, *n* (%)	60 (92)		
Current/previous smoker, *n* (%)	28 (43.1)		
Hypercholesterolaemia, *n* (%)	15 (23.1)		
Hypertension, *n* (%)	19 (29.2)		
Admission HR, beats per minute	76 ± 12		
Admission Systolic BP, mmHg	124 ± 26		
Admission Diastolic BP, mmHg	75 ± 15		
Peak Creatine Kinase, iU/L	1064 (418–2588)		
Anterior STEMI, *n* (%)	30 (46)		
Discharge Medications			
Aspirin, *n* (%)	57 (88)		
Clopidogrel, *n* (%)	59 (91)		
Warfarin, *n* (%)	6 (9)		
Statin, *n* (%)	63 (97)		
Beta-blocker, *n* (%)	64 (99)		
ACEi/ARA, *n* (%)	63 (97)		
Loop/Thiazide Diuretic, *n* (%)	5 (8)		
Spironolactone/Eplerenone, *n* (%)	7 (11)		
CMR Parameters	Baseline	Follow-up	*p*-value
LVEDVI, ml/m^−2^	91.1 (84.5–102.2)	93.5 (85.0–106)	0.454
LVESVI, ml/m^−2^	53.5 (47.6–65.9)	47.7 (39.8–61.6)	0.001
LVEF, %	41.0 ± 8.40	47.2 ± 8.46	<0.001
IS, % (of LV mass)	22.3 (14.5–35.5)	17.0 (12.3–22.8)	<0.001

*Abbreviations*: *ACEi* (Angiotensin Converting Enzyme Inhibitor), *ARB* (Angiotensin-II Receptor Blocker), *BP* (Blood Pressure), *CAD* (Coronary Artery Disease), *HR* (Heart Rate), *IS* (Infarct Size), *LV* (Left Ventricular), *LVEDVI* (Left Ventricular End Diastolic Volume), *LVEF* (Left Ventricular Ejection Fraction), *LVESVI* (Left Ventricular End Systolic Volume), *N/A* (Not Applicable), *STEMI* (ST-segment Elevation Myocardial Infarction)

### Cmr findings

Key patient characteristics are shown in Table 1. Compared with baseline, there was a decrease in LV end-systolic volume index at follow up (47.7 ml.m^−2^ versus 53.5 ml.m^−2^, mean difference −5.6 ml.m^−2^, *p* < 0.001) and an increase in LVEF (47.2 ± 8.5 versus 41.0 ± 8.4%, mean difference 6.2%, *p* < 0.001). Eleven (16.9%) patients had developed adverse LV remodelling – nine (13.8%) patients had an increase in LV end-systolic volume index ≥15%, and six (9.2%) patients had an increase in LV end-diastolic volume index ≥20%.

### Strain at baseline

Strain analysis was possible in all (*n* = 65) patients with FT and in 64 patients with tagging (one patient had non-analysable images). GCS was significantly higher with FT than with tagging (−21.1 ± 6.3% versus −12.1 ± 4.3%, *p* < 0.001). GLS by FT was −13.2 ± 5.5%.

### Correlation of strain with baseline lvef & is

There was good correlation of GCS, measured by both FT and tagging, with baseline IS (*ρ* = −0.72 for tagging and *ρ* = −0.61 for FT) and LVEF (*r* = 0.70 for tagging and *r* = 0.71 for FT). GLS (FT) also had similar correlation with baseline IS and LVEF (Table 2).

**Table 2:** Correlation of baseline strain parameters with LVEF and IS

	Infarct size (IS)	Ejection Fraction (LVEF)
Tagging (*n* = 64)		
GCS	−0.72**	0.70**
Feature Tracking		
GCS	−0.61**	0.71**
GLS	−0.62**	0.64**

*Abbreviations: EF* (Ejection Fraction), *GCS* (Global Circumferential Strain), *GLS* (Global Longitudinal Strain), *IS* (Infarct Size)

Note: Spearman’s Rank coefficient (ρ) for IS, Pearson’s correlation coefficient (r) for EF

**p < 0.01

### Prediction of adverse lv remodelling

**Fig. 3:**
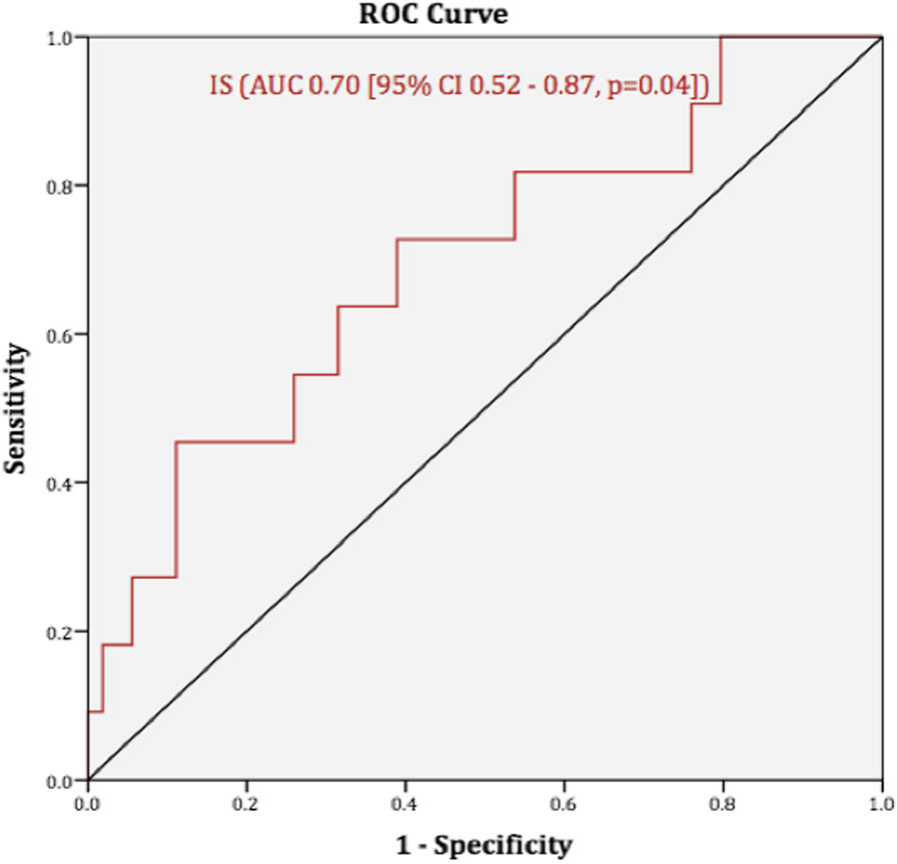
Receiver Operator Characteristic *Curve* for baseline Infarct Size to predict LV End-Systolic Volume Index ≥15% at follow-up versus baseline. Abbreviations: AUC (Area Under the *Curve*); CI (95% Confidence Interval); IS (Infarct Size)

None of the baseline strain parameters was able to predict the development of adverse LV remodelling (Table 3). Only baseline IS predicted the development of adverse LV remodelling with statistical significance, albeit modestly – Odds Ratio 1.05 (1.01–1.10, *p* = 0.03, i.e. the odds of a patient having developed adverse LV remodelling increased by 0.05% for every unit increase in baseline IS). In predicting adverse remodelling, the area-under-the curve with receiver operator characteristic curve analysis for baseline IS was moderate – 0.70 (0.52–0.87, *p* = 0.04) (Fig. 3).

**Table 3:** Global Strain and IS to predict development of LV remodelling

Baseline Variable	Prediction of Adverse Remodelling – OR (95% CI, *p*-value)
GCS (FT), %	0.92 (0.83–1.03, *p* = 0.16)
GLS (FT), %	0.90 (0.79–1.03, *p* = 0.14)
GCS (Tagging), %	0.88 (0.74–1.05, *p* = 0.15)
IS, %	1.05 (1.01–1.10, *p* = 0.03)*

*Abbreviations: CI* (Confidence Interval), *FT* (Feature Tracking), *GCS* (Global Circumferential Strain), *GLS* (Global Longitudinal Strain), *IS* (Infarct Size) *OR* (Odds Ratio)

*p < 0.05

## Discussion

This is the first study to evaluate the role of CMR-based strain assessment (using both tagging and FT) in predicting adverse LV remodelling following STEMI. Our data showed that although global strain parameters appear to have a good correlation with baseline IS, none of them can significantly predict the development of remodelling. The only significant predictor of adverse LV remodelling appears to be baseline IS and this is consistent with previous results [7].

The only other study to evaluate the role of CMR-based global strain at predicting endpoints post-STEMI evaluated FT in isolation (without tagging) and showed that GCS was associated with the development of LVEF > 50% at follow-up [15]. However, this study identified ‘low risk’ subjects with global functional LV recovery and rather than those with adverse LV remodelling.

Previous studies have shown that strain, as evaluated by both tagging and FT, appears to be decreased in the myocardial segments in the infarcted region compared with non-infarcted segments [21, 22]. This may explain the good correlation between baseline strain parameters and IS. However, unlike IS, which is purely a measure of myocardial scar burden, global strain is influenced by both infarct- and non-infarct-related myocardial segments. Consequently, there may have been hyperkinetic wall motion in the non-infarct related segments leading to a ‘preserved’ overall global strain value in some patients. This may explain the superiority of IS in predicting adverse LV remodelling given that, unlike global strain, it is purely a measure of the extent of myocardial damage post-infarction. Furthermore, segmental strain analysis by both tagging and FT has been shown to have high intra- and inter-observer variability and therefore may not be a reliable measure of LV function in a clinical setting [14].

Our results suggest that CMR-based global strain may not have a significant role to play in the setting of acute STEMI as has previously been suggested [15]. Larger studies will be required to determine whether acutely measured strain by CMR is predictive of clinical endpoints (such as mortality and/or development of Major Adverse Cardiac Events, MACE) and provides incremental prognostic data compared with IS assessment alone.

### Limitations

This study had a relatively small sample size with a small proportion of patients developing remodelling. This may partly represent the beneficial effects of secondary prevention measures (angiotensin-converting enzyme inhibitors, beta-blockers and statins) which were prescribed in the majority of patients and known to prevent remodelling post-STEMI [23]. Additionally, other markers of poor post-STEMI outcomes, such as age and infarct location, could not be accounted for in the regression model without risk of ‘over-fitting’ [24, 25]. Our patients were predominantly male so further studies are required in cohorts with larger numbers of female patients [26]. Technical limitations of CMR-based strain assessment include sub-optimal tracking of endocardial motion on some post-contrast SSFP images due to reduced contrast-to-noise ratio between the blood pool and myocardium (FT) and image degradation due to poor breath-holding and ectopy (tagging) [14]. Tagging sequences were not acquired in long axis views and hence the value of GLS after STEMI could not be assessed.

## Conclusions

CMR measured global strain was correlated with baseline IS following STEMI but had no significant value in predicting adverse LV remodelling. Further work is needed to determine if this holds true for the prediction of clinical endpoints post-STEMI.

## Abbreviations

CMR: Cardiac magnetic resonance imaging
FT: Feature tracking
GCS: Global circumferential strain
GLS: Global longitudinal strain
IS: Infarct size
LV: Left ventricular
LVEF: Left ventricular ejection fraction
PCI: Percutaneous coronary intervention
SAX: Short axis
STEMI: ST-segment elevation myocardial Infarction.
